# Investigating the effects of formative assessment on EFL students’ achievement and motivation: a Self-Determination Theory perspective

**DOI:** 10.3389/fpsyg.2025.1664871

**Published:** 2025-12-12

**Authors:** Han Zhang, Jiale Quan, Ning Zhang, Lingyu Zhang

**Affiliations:** 1 Hebei Minzu Normal University, Chengde City, Hebei Province, China; 2 Hebei Normal University, Shijiazhuang City, Hebei Province, China; 3 University of Malaya, Kuala Lumpur, Malaysia; 4 Shaanxi Institute of Technology, Xi’an City, Shaanxi Province, China; 5 Taylor’s University, Kuala Lumpur, Malaysia

**Keywords:** Self-Determination Theory, formative assessment, English as a foreign language, motivation, autonomy, relatedness, competence

## Abstract

**Introduction:**

This quasi-experimental study investigated whether formative assessment enhances EFL students’ motivation and English achievement. Drawing on Self-Determination Theory, which highlights autonomy, competence and relatedness as core psychological needs, the study examined how formative assessment may satisfy these needs to improve learning motivation.

**Methods:**

Eighty-nine middle school EFL students in China participated in an eight-week quasi-experimental intervention. One group received formative-assessment-based instruction, while the comparison group experienced conventional teaching. Data were collected through SDT-based motivation questionnaires and English achievement tests.

**Results:**

Findings showed that formative assessment most strongly enhanced students’ sense of relatedness, potentially due to closer peer and teacher relationships and greater enjoyment of English learning. Improvements in autonomy and competence were present but less substantial. Regression analysis further demonstrated that autonomy was the strongest predictor of English achievement, indicating its key role in linking motivational resources to learning outcomes.

**Discussion:**

The study highlights the differential effects of formative assessment on students’ psychological needs and underscores the importance of autonomy in academic achievement. Several limitations are acknowledged, including the small sample size, single-school setting, brief intervention period, and possible cultural or teacher-related influences, all of which may constrain generalisability. Future research should adopt longitudinal designs, recruit more diverse samples, and investigate how specific formative assessment strategies uniquely support autonomy, competence and relatedness.

## 1 Introduction

Assessment, as a crucial component of the learning process, plays an essential role in enhancing student learning by helping them set goals, monitor progress, reflect on their learning, and receive feedback that promotes further development (Luo et al., 2020). While summative assessment (SA) focuses on evaluating outcomes at the end of instruction, formative assessment (FA) is conceptualized as a set of continuous classroom practices wherein teachers, students, or peers elicit, interpret, and use evidence of learning to inform subsequent instruction (Black and Wiliam, 2009). This process is intended to improve both teaching and learning by making decision-making more responsive and evidence-based (Bennett, 2011).

However, implementing FA in Chinese middle school EFL (English as a foreign language) classrooms poses unique challenges. The educational environment in China is often examination-oriented and teacher-centered, where English learning is heavily driven by high-stakes testing (Chen et al., 2021). These conditions tend to suppress learner autonomy and reduce the opportunities for interactive, student-centered assessment. According to Zhang et al. (2024), while recent studies have explored peer assessment, self-assessment, portfolios, feedback, and questioning in EFL contexts, only 15% of the research implemented FA holistically, and few have examined its psychological mechanisms in depth. As a result, FA remains underutilized despite its documented benefits in China.

Although studies report mixed outcomes regarding the effectiveness of FA on language learning, such as speaking comprehension (e.g., Chien et al., 2020), writing ability (e.g., Xie and Lei, 2019), self-regulated learning (e.g., Xiao and Yang, 2019), vocabulary learning (e.g., Nourdad and Banagozar, 2022) and reading comprehension (Ahmadi et al., 2021), they consistently highlight the critical role of learner motivation. Motivation not only affects how students respond to assessment but also influence whether they engage with feedback, reflect on their progress, or take ownership of their learning. Surprisingly, many EFL teachers remain reluctant to adopt FA practices due to limited assessment literacy and lack of training (Zhang et al., 2024), despite recent efforts to improve professional development in this area (Peter Gu and Lam, 2023).

To better understand how FA may foster motivation, this study draws on Self-Determination Theory (SDT), which posits that three basic psychological needs: autonomy, competence, and relatedness, must be fulfilled to support intrinsic motivation and sustained learning engagement (Ryan and Deci, 2020). Many researchers have found that FA closely aligns with these needs proposed by SDT (Pat-El et al., 2024; Lin, 2025). For example, autonomy is supported when students are actively involved in their own learning through activities such as self-assessment and goal-setting, which enable them to make meaningful choices and take ownership of their progress. Competence is fostered through constructive feedback and scaffolded tasks that provide clear indicators of success and help students perceive improvement over time (De Jonge et al., 2025; Dumitru and Dragomir, 2025). Relatedness is addressed when peer assessment and collaborative learning opportunities are embedded in the classroom, allowing students to feel connected to both their classmates and teacher (Yilmaz Soylu et al., 2025; Baines et al., 2025). These principles suggest that well-designed FA practices may not only enhance academic achievement but also strengthen motivational foundations by fulfilling psychological needs (Pat-El et al., 2024; Jaison et al., 2025).

While specific FA practices are theorized to promote learners’ motivation by supporting three needs, there is still limited empirical evidence on how a cohesive FA framework, when implemented systematically in classroom instruction, influences the motivational process. In particular, in exam-driven EFL classrooms in China, where students often have limited agency and engagement, it remains unclear whether a structured FA intervention can effectively address students’ psychological needs and, in turn, their academic achievement. Rather than isolating individual FA strategies, the present study adopts an integrated FA approach that combines goal setting, reflection, peer/self-assessment, and formative feedback, delivered over an eight-week period. This design aims to provide a more ecologically valid understanding of how FA functions in authentic EFL teaching and how student motivation may mediate the relationship between FA and students’ academic achievement.

## 2 Literature review

### 2.1 FA in EFL classroom

Various empirical studies have examined the impact of FA practices on EFL education. These studies have explored a range of learning outcomes, including speaking performance (Chien et al., 2020), test anxiety (Contreras-Soto et al., 2019), reading and writing ability (Liu and Brantmeier, 2019), conceptions of writing (Gebrekidan and Zeru, 2023) and psychological factors such as academic motivation, attitude toward learning, test anxiety, and self-regulation skill (Ismail et al., 2022). Collectively, these findings underscore the role of FA in helping students identify their weaknesses and direct their effort toward areas needing improvement.

Recent research has also examined the implementation of FA interventions in pedagogical contexts. For instance, Ahmadi et al. (2021) applied FA techniques such as reading questions (RQs), to address learners’ persistent difficulties in reading comprehension and vocabulary use. Their results indicated that RQs, as a formative strategy, facilitated improved comprehension and vocabulary retention by increasing learner interaction, offering communicative feedback, and providing frequent opportunities for contextualized practice. Similarly, Weng et al. (2023) found that FA had significant effects on students’ attitudes toward EFL writing courses and substantially improved their self-confidence in writing.

In the Chinese context, several studies have highlighted the benefits of FA. Liu and Brantmeier (2019) found that FA scores in reading and writing were significantly correlated with standardized test results among young Chinese EFL learners, reinforcing the validity of FA as an instructional approach. Similarly, Xiao and Yang (2019) reported that FA activities, particularly when paired with feedback, enabled learners to set goals, interpret and act on feedback, manage learning resources, and take meaningful actions to advance their learning, thereby developing stronger self-regulatory capacity.

In parallel, a growing number of studies have investigated FA from the perspective of EFL teachers, focusing on their beliefs (e.g., Vattøy, 2020), perceptions (e.g., Arefian, 2022) and FA literacy (e.g., Bustamante, 2022). Zeng and Huang (2021), for instance, observed that many teachers had only a superficial understanding of FA and that their classroom assessment practices were often limited in scope. According to teachers’ perceptions, FA can positively impact students’ self-regulated learning, motivation and reflective ability. While many teachers acknowledged the value of FA in promoting student motivation, self-regulation, and reflection (Vogt et al., 2020), they also cited implementation barriers, including insufficient training, limited feedback strategies, and underutilization of digital tools (Mäkipää, 2021).

In summary, FA plays a pivotal role in shaping both the processes and outcomes of EFL teaching and learning. It directly contribute to students’ motivation, engagement, and learning autonomy, while also influencing teachers’ instructional practices. A more nuanced understanding of how FA affects learners, particularly through motivational pathways, is essential for designing effective, supportive, and context-sensitive EFL learning environments.

### 2.2 Self-Determination Theory in EFL learning

Motivation is widely regarded as a central determinant of success in language learning (Dörnyei and Ushioda, 2021). It functions as a cognitive and emotional engine that drives effort, persistence, and enjoyment in learning activities (Gardner, 2006). In the context of EFL education, motivation plays a pivotal role in shaping learning behaviors and academic outcomes (Guilloteaux and Dörnyei, 2008). For instance, Guo and Bai (2022) found that learners with higher levels of motivation tended to achieve better English language proficiency, whereas those with lower motivational engagement often demonstrated weaker academic performance. A lack of motivation may lead to disengagement, reduced effort, and, ultimately, failure to acquire the target language effectively.

To better understand the nature of motivation in educational contexts, SDT offers a comprehensive and empirically supported framework (Deci and Ryan, 2000). SDT posits that the satisfaction of three innate psychological needs: autonomy, competence, and relatedness, is essential for optimal motivation, wellbeing, and learning. According to Ryan and Deci (2020), teachers play a vital role in shaping classroom environments that either support or hinder the fulfillment of these needs. When students perceive that these needs are met, they are more likely to demonstrate intrinsic motivation, deeper engagement, and greater academic achievement.

SDT has increasingly been applied in EFL research to explore how psychological needs influence language learning. Vadivel et al. (2022), for instance, examined the roles of autonomy, competence, relatedness, and anxiety, finding that satisfaction of these needs significantly improved learners’ engagement and satisfaction in English classrooms. Conversely, unmet needs were associated with diminished motivation and lower wellbeing. Al-Abidi et al. (2023) explored the influence of mobile applications on EFL motivation and reported marginal improvements in autonomy and competence, though no notable gains in relatedness were observed. These studies highlight the contextual variability of motivational outcomes, depending on how learning environments address each SDT components.

Among the three needs, autonomy refers to the learner’s experience of volition and self-direction in learning. Several studies (Aiusheeva and Guntur, 2019; Noels et al., 2019) affirm that when learners feel autonomous, they tend to take greater ownership of their language development. Mobile-assisted language learning tools have been shown to promote this sense of autonomy. For example, Yan et al. (2023) found that the use of mobile learning platforms enhanced learners’ autonomous motivation and academic performance. Similarly, Alamer and Al Khateeb (2023) noted that WhatsApp-based instruction reduced anxiety and fostered autonomy in EFL learners. Competence, the second SDT need, reflects students’ perception of their ability to succeed in learning tasks. When learners feel competent, they are more likely to engage in challenging activities and persevere through difficulties (Ryan and Deci, 2020). Conesa et al. (2022) argued that competence arises when students receive clear feedback, achieve task success, and observe tangible progress. Shelton-Strong (2022) demonstrated that reflective dialogues, often used as part of FA strategies, significantly enhanced learners’ perceived competence and motivation. Relatedness refers to the need for connection, belonging, and meaningful relationships (Ryan and Deci, 2020). In EFL classrooms, relatedness is fostered through peer collaboration, social engagement, and teacher–student interaction. Escandell and Chu (2023) identified four strategies for enhancing relatedness in language classrooms: fostering interpersonal connections, reducing learner silence, facilitating reciprocal feedback, and promoting student-centered pedagogy. Their findings suggest that classroom environments that nurture relatedness contribute to higher learner motivation and improved language outcomes.

The literature indicates that SDT provides a valuable lens for understanding motivational processes in EFL learning. When classroom conditions support autonomy, competence, and relatedness, learners are more likely to engage deeply, regulate their own learning, and ultimately achieve better academic outcomes. These insights underscore the potential to integrate SDT-informed practices into EFL pedagogy, particularly through the use of FA strategies that operationalize psychological need satisfaction.

### 2.3 FA and SDT

**Figure 1:**
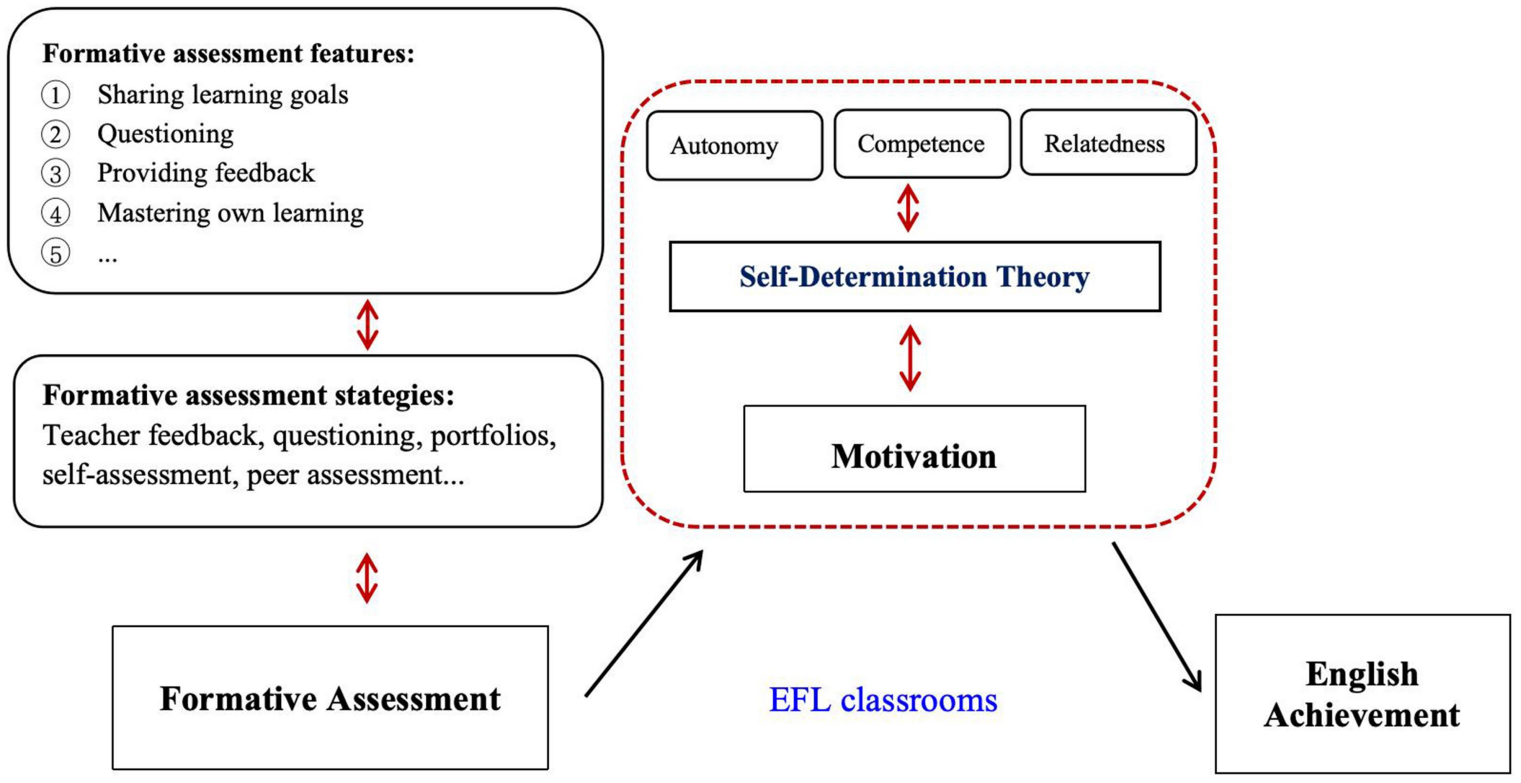
Conceptual framework of the study.

Existing research consistently indicates that FA is not only a means of monitoring learning but also a teaching intervention that can significantly stimulate student motivation (Lin, 2025). According to SDT, students’ intrinsic motivation is driven by three basic psychological needs: autonomy, competence, and relatedness (Ryan and Deci, 2020). Specific FA practices, such as feedback, reflection, and self- and peer-assessment, enhance student motivation by satisfying these needs. The conceptual framework for this study (see Figure 1) illustrates how FA practices may activate these motivational mechanisms, thereby influencing EFL achievement.

FA has been widely demonstrated to effectively enhance students’ sense of competence. For example, De Jonge et al. (2025), in field research at the secondary school level, found that providing goal clarification and process feedback before low-stakes tests reduced students’ test anxiety. This effect was primarily achieved by enhancing students’ positive perceptions of their own abilities. Furthermore, Pat-El et al. (2024) noted that students’ perceptions of FA can further enhance their intrinsic learning motivation by satisfying their needs for competence and autonomy.

Additionally, self-assessment and reflection tasks provide students with opportunities to take control of their learning, thereby fulfilling the need for autonomy. In comparing extensive and intensive reading instruction, Lin (2025) found that encouraging students to reflect on and evaluate their reading improved both self-regulation and reading motivation. Similarly, in a university accounting course, Dumitru and Dragomir (2025) used a “student-generated questions” strategy to promote active engagement with content, significantly enhancing their autonomy and sense of competence. Wang et al. (2024) further noted that students’ positive perceptions of FA can enhance their learning autonomy by promoting psychological empowerment and positive academic emotions, demonstrating a chain-mediation effect.

Peer assessment and collaborative assessment activities also contribute to the satisfaction of relatedness needs. Although peer assessment has received less study in this literature, the group assessment and collaborative problem-solving process introduced by Dumitru and Dragomir (2025) enabled students to interact and support one another, fostering a sense of classroom community and mutual recognition, factors known to reinforce sustained motivation. Zhang and Crawford (2024) found that gamified FA, featuring elements such as badges, leaderboards, and progress tracking, promoted autonomy by fostering ownership of learning and competence through clear achievement indicators. Simultaneously, the competitive and cooperative dynamics of the games supported social engagement, thus fulfilling the need for relatedness.

Furthermore, FA strategies in digital environments demonstrate unique motivational potential. Lin et al. (2025) found that gamified assessment activities were most effective when tasks were perceived as both challenging and engaging, fulfilling the need for competence through the experience of flow. Meanwhile, Yilmaz Soylu et al. (2025) noted that the AI-assisted assessment tool “Socratic Mind” enhances students’ autonomy through personalized feedback and interactive processes. Similarly, Nikou and Economides (2021) reported that mobile-assisted FA enhanced learners’ sense of autonomy, competence, and relatedness by providing immediate feedback, allowing students to track their own progress, adjust their learning pace, and collaborate with peers. Chemsi et al. (2020) also found that digital FA systems embedded in Moodle empowered students to continuously monitor learning processes and receive teacher feedback, thereby improving motivation through greater autonomy.

Collectively, the three basic psychological needs identified in SDT: autonomy, competence, and relatedness, serve as pivotal constructs in the study of EFL learning. Building on this foundation, the present research aims to extend the literature by exploring (a) how formative assessment influences these SDT-based motivational variables, and (b) how these motivational variables mediate the impact of FA interventions on learners’ EFL achievement.

*RQ1:* Is there a significant disparity in scores within motivation domains for the EG and CG, after controlling the results from their first test and second test results?

*RQ2:* Does motivation mediate the effect of FA on EFL achievement, and which motivational factor contributes most strongly to this pathway?

## 3 Research design

### 3.1 Participants

The participants in this study were 89 EFL students from a public middle school (referred to as Middle School A), in Chengde city, Hebei province, China. The school follows both national and provincial policies, which facilitated the implementation of diverse FA strategies essential to this study. Additionally, students came from various socio-economic backgrounds, providing a rich context for examining the effect of FA on EFL motivation and achievement within a heterogeneous student population. The participants were between 13 and 15 years old, and English is a compulsory subject in their curriculum.

According to Jack et al. (2012), fully randomized experimental designs may compromise ecological validity by disrupting natural classroom settings, such as breaking up regular class arrangement. Conversely, using an intact class in educational research offers findings that are more transferable to real-world classroom settings (Creswell and Creswell, 2023). Therefore, this study adopted a quasi-experimental, non-randomized design using intact class assignment and cluster sampling to maintain classroom authenticity and instructional continuity.

Initially, the researcher contacted 22 EFL teachers at Middle School A to determine their willingness to participate in the experiment. All six EFL teachers from the seventh grade agreed. Among the seventh-grade classes, Classes 1 and 2 were selected as the experimental group (EG) and control group (CG), respectively, because they were taught by the same teacher and had similar academic performance and socio-economic backgrounds, ensuring baseline comparability for the quasi-experimental design.

The remaining eight classes (a total of 407 students) participated in the pilot study. In total, 89 EFL students from classes 1 and 2 were included in the main study. Class 1 comprised 23 female and 22 male students, while Class 2 comprised 22 female and 23 male students. The two classes were comparable in academic performance, gender ratio, and instructional time. All students had begun learning English in the third grade (around age nine).

### 3.2 Instruments

This study used two research instruments: a motivation questionnaire and a set of English achievement tests. The motivation survey, adapted from Chen et al. (2015), assessed the psychological needs of autonomy, relatedness, and competence, as defined in SDT. English achievement was measured through three tests administered during the first, fourth and eighth weeks to capture changes over the intervention period.

The adapted questionnaire measured participants’ psychological needs in the EFL context. Modifications were made to the original items to ensure their relevance to English language learning. The final version contained 24 items (e.g., *“I feel excluded from the group I want to belong to in EFL classes”*), rated on a 5-point Likert scale ranging from *strongly disagree* to *strongly agree*. The reliability of the full scale, calculated using Cronbach’s alpha, was 0.976. Subscale reliabilities were also high: autonomy = 0.953, competence = 0.893, and relatedness = 0.950, indicating excellent internal consistency.

To homogenize students’ general English proficiency, a version of the official standardized test, adapted from exams administrated by the local education bureau, was administered. Due to time and logistical constraints, only the reading, grammar, and writing sections were included. The test was piloted on a comparable group, with a 45-min time limit. Its content validity was confirmed by subject experts, and its reliability coefficients were acceptable (Cronbach’s alpha = 0.70; KR-20 = 0.70).

### 3.3 Intervention plan

The FA-based intervention comprised 12 structured lessons, each lasting 40 min, delivered over an eight-week period. Each lesson incorporated at least one FA activity, such as peer assessment, self-assessment, reflective writing, or teacher feedback. To ensure instructional consistency, 12 standardized lesson plans were developed and implemented across all intervention classes.

To prepare for the intervention, all 22 English teachers at Middle School A participated in a 10-day training program on FA. From this group, six seventh-grade EFL teachers agreed to take part in the study. Since Classes 1 and 2 were taught by the same teacher and had comparable academic performance and socioeconomic backgrounds, they were designated as the EG and CG, respectively, using cluster-randomized sampling procedures. The remaining eight seventh-grade classes (*n* = 407) participated the pilot study to test instruments and refine intervention materials. A standardized set of lesson plans and instructional materials was used by all participating teachers to ensure consistency and fidelity in delivering the intervention.

Throughout the intervention, participating teachers received ongoing support via weekly coordination meetings to maintain implementation fidelity. Appendix A outlines the FA components embedded in each lesson, including assessment focus, tools used (e.g., rubrics, checklists), and frequency. Appendix B presents two sample lesson plans (reading and writing), detailing objectives, FA activities, and assessment strategies. For example, following Lesson 1 (reading), students completed a recall task and peer assessment using a chart template. In Lesson 2 (writing), students created imaginative content, which was peer-reviewed using a writing rubric.

Teacher fidelity to the intervention was monitored through classroom observations and teacher logs. Observers used a checklist (developed during the pilot study) to verify whether FA strategies (e.g., peer assessment) were implemented as intended. Additionally, teachers submitted weekly self-reports to document FA usage and reflect on implementation challenges.

### 3.4 Data collection procedure

At the start of the intervention, all students completed an initial motivation questionnaire and an English achievement test to establish baseline measures. These instruments were administered again in the fourth and eighth weeks to track changes over time. During Week 1, the teacher introduced FA practices and began delivering the designed lesson plans for both groups.

The control group received conventional instruction, which included the grammar-translation method, task-based language teaching, and SA, such as tests without formative feedback. Instruction followed the standard curriculum, and evaluations were conducted primarily through end-of-unit tests and exams. In contrast, the experimental group received an intervention integrating FA practices into their regular learning. This included continuous feedback to guide improvement, as well as self-assessment and peer-assessment activities to support reflection and individualized progress. Instruction was adjusted based on ongoing assessment data. Table 1 summarizes the procedures for both groups, detailing the conventional teaching approach used with CG and the FA strategies integrated into the EG’s instruction. In the CG, the assessment is aimed primarily at assigning grades, with evaluators relying solely on fixed standards. In contrast, the EG focused on promoting learning, with evaluation involving a more complex process of identifying performance gaps, analyzing underlying causes, and determining strategies to address them. During the whole procedure, no students withdrew from the study, and all questionnaire responses were valid and included in the final analysis.

**Table 1:** Features between CG and EG.

CG	EG
When: At the end of the lesson or after the learning, no more formal learning is occurring at this stage; Purpose: to measure or summarize what students have learned; How: use information collection tools to make judgments about the students. They are meant to grade different language skills and learners’ achievement; Main role: Evaluate EFL students’ achievement; Use of judgment results: report.	When: During each lesson. Purpose: supply information to be utilized as feedback to adjust and facilitate the learning and teaching activities; How: use information collection tools to compare the actual level with the standard level and determine the gap; Determine the cause of the gap; Determine improvement measures, provide feedback; Main role: Observe EFL students’ learning and provide feedback based on their weaknesses and strengths; Use of judgment results: Try improvement measures.

### 3.5 Data analysis

This study employed Multivariate Analysis of Covariance (MANCOVA) and multiple regression analysis to examine the research questions. MANCOVA was used to compare group differences in motivational domains while statistically controlling for baseline scores (i.e., relatedness 1, autonomy 1, and competence 1), consistent with best practices in quasi-experimental design. Subsequently, a multiple regression analysis was conducted to test whether the three motivational variables, autonomy, competence, and relatedness, mediated the relationship between formative assessment and EFL achievement outcomes.

Prior to these analyses, all necessary statistical assumptions were checked. For MANCOVA, homogeneity of covariance matrices was assessed using Box’s M Test, and Wilks’ Lambda (*p* < 0.05) was used as the multivariate test statistic (Pallant, 2020). Motivation scores from Time 1 (T1) and Time 2 (T2) were included as covariates in the final model. For multiple regression, assumptions of normality, linearity, homoscedasticity, and independence of residuals were assessed using P–P plots and scatterplots. Multicollinearity was examined via correlation coefficients (*r* ≥ 0.90) and Variance Inflation Factors (VIF > 10). In addition, Durbin-Watson statistics were checked to assess autocorrelation in the residuals. All analyses were conducted using SPSS. Effect sizes and 95% confidence intervals were reported for all tests. For MANCOVA, partial eta squared (*η*^2^) values were used to indicate the magnitude of group differences. For the regression models, standardized beta coefficients (*β*) and significance levels (*p* < 0.05) were reported to identify the contribution of each motivational variable. Where applicable, *R*^2^ and adjusted *R*^2^ values were reported to indicate model fit.

## 4 Findings

### 4.1 Result of RQ1

**Figure 2:**
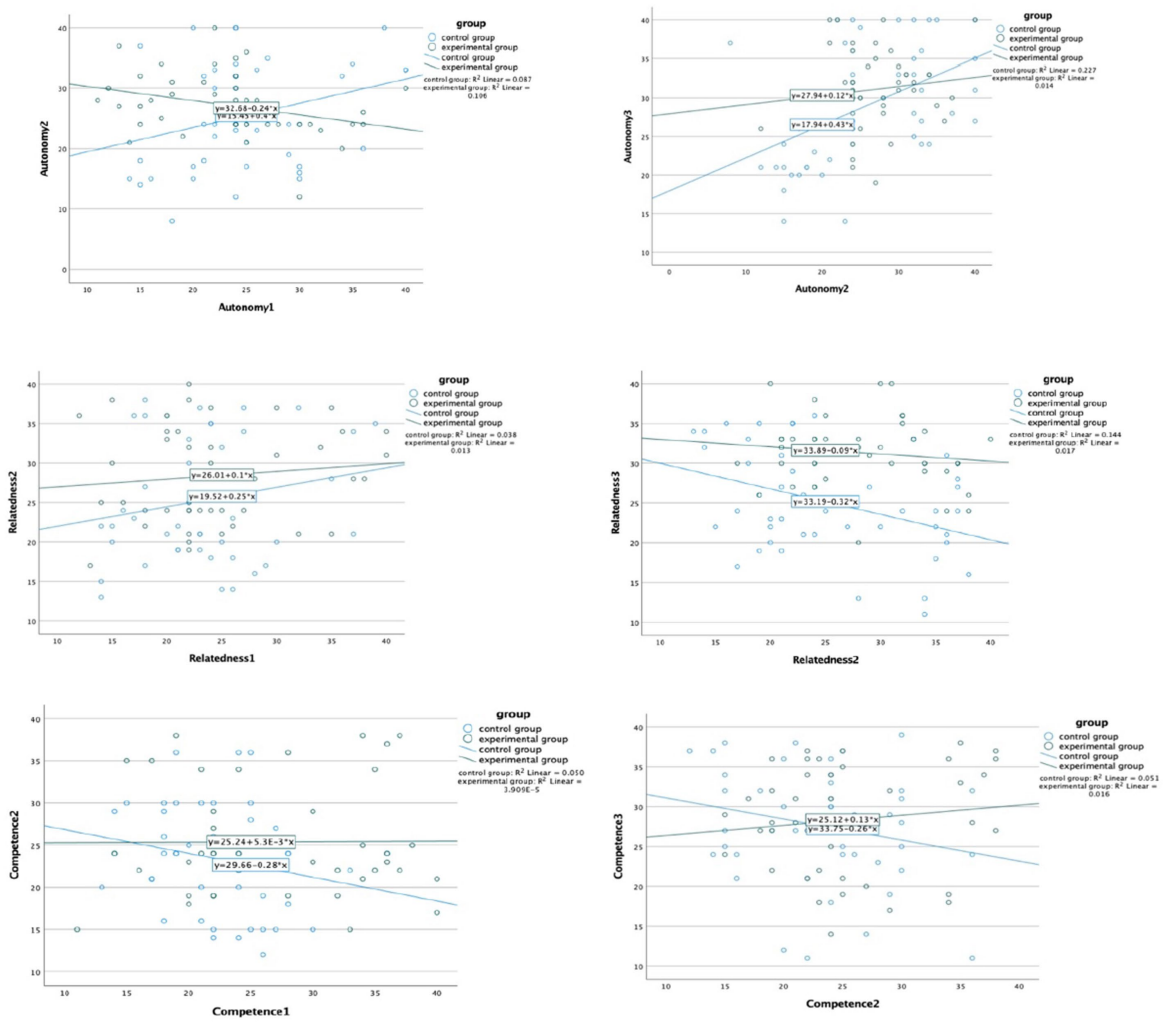
Scatterplots between two Groups in the three times autonomy, competence, relatedness scores.

Before performing the analyses, normality and outliers were checked. Table 2 presents descriptive statistics, including the means, standard deviations, and normality test based by skewness and kurtosis. Additionally, Scatterplots were manually checked for linear, and the points generally appeared to follow the line (see Figure 2). These results suggest the data are normally distributed.

**Table 2:** The descriptive statistics and normality test of motivation domain scores and achievements.

Group			N statistic	Mean statistic	Std. Deviation statistic	Skewness	Kurtosis
CG	First week	Autonomy 1	45	24.93	6.362	0.638	0.321
		Relatedness 1	45	23.27	6.181	0.710	0.393
		Competence 1	45	22.42	4.943	0.335	0.256
	Fourth week	Autonomy 2	45	25.47	8.657	0.045	−1.073
		Relatedness 2	45	25.27	7.785	0.281	−1.338
		Competence 2	45	23.33	6.263	0.126	−0.590
	Eighth week	Autonomy 3	45	28.84	7.787	−0.003	−1.153
		Relatedness 3	45	25.09	6.574	−0.041	−0.705
		Competence 3	45	27.58	7.319	−0.570	−0.090
EG	First week	Autonomy 1	44	23.32	7.060	0.283	−0.351
		Relatedness 1	44	24.57	7.296	0.555	−0.324
		Competence 1	44	26.91	7.709	−0.002	−1.049
	Fourth week	Autonomy 2	44	27.16	5.131	0.073	1.142
		Relatedness 2	44	28.41	6.177	0.125	−1.172
		Competence 2	44	25.39	6.535	0.664	−0.555
	Eighth week	Autonomy 3	44	31.14	5.019	−0.218	0.015
		Relatedness 3	44	31.32	4.236	−0.057	0.582
		Competence 3	44	28.34	6.548	−0.354	−0.893

Before conducting the MANCOVA, the assumption of homogeneity of regression slopes was examined. This test evaluates whether the relationship between the covariates and the dependent variables is consistent across groups. As shown in Tables 3, 4, the assumption was largely satisfied. For the control group, all interaction effects were non-significant (*p* > 0.05). For the experimental group, most interactions were also non-significant (*p* > 0.05), except for Relatedness 2, where a significant effect was observed (*p* = 0.005). This suggests that the assumption was generally met. Additionally, Levene’s tests (Table 5) indicated that the assumption was violated for Autonomy 2, Autonomy 3 and Relatedness 3 (*p* < 0.05). The assumption was met for Relatedness 2, Competence 2, and Competence 3 (*p* > 0.05). Consequently, findings relating to autonomy and relatedness should be interpreted with caution.

**Table 3:** Homogeneity of regression slopes for the second motivation domain and the first motivation domain.

Tests of between-subjects effects							
Group	Source	Dependent variable	Type III sum of squares	df	Mean square	*F*	Sig.
CG	Group * Autonomy 1 * Relatedness 1 * Competence 1	Autonomy 2	0.036	1	0.036	0.001	0.981
		Relatedness 2	97.557	1	97.557	1.614	0.211
		Competence 2	22.258	1	22.258	0.586	0.449
EG	Group * Autonomy 1 * Relatedness 1 * Competence 1	Autonomy 2	30.170	1	30.170	1.230	0.274
		Relatedness 2	299.410	1	299.410	8.900	0.005
		Competence 2	126.752	1	126.752	3.741	0.060

**Table 4:** Homogeneity of regression slopes for the second motivation domain and the third motivation domain.

Tests of between-subjects effects							
Group	Source	Dependent variable	Type III sum of squares	df	Mean square	*F*	Sig.
CG	Group * Autonomy 2 * Relatedness 2 * Competence 2	Autonomy 3	30.220	1	30.220	0.619	0.436
		Relatedness 3	58.432	1	58.432	1.516	0.225
		Competence 3	32.872	1	32.872	0.671	0.417
EG	Group * Autonomy 2 * Relatedness 2 * Competence 2	Autonomy 3	79.150	1	79.150	3.338	0.075
		Relatedness 3	0.544	1	0.544	0.030	0.864
		Competence 3	0.478	1	0.478	0.010	0.919

**Table 5:** Levene’s test of equality of error variances in the second and the third motivation domains.

Dependent variables	*F*	df1	df2	Sig.
Autonomy 2	9.332	1	87	0.003
Relatedness 2	2.955	1	87	0.089
Competence 2	0.241	1	87	0.625
Autonomy 3	6.144	1	87	0.015
Relatedness 3	9.063	1	87	0.003
Competence 3	0.029	1	87	0.864

When the first motivation domain (Autonomy 1, Relatedness 1, and Competence 1) was entered as covariates, the multivariate effect of group did not reach statistical significance, Pillai’s Trace is 0.093 (>0.05), partial *η*^2^ = 0.075 (see Table 6). This indicates that FA did not produce a significant overall difference in the combined dependent variables (Autonomy 2, Relatedness 2, Competence 2) after controlling for baseline motivation. Follow-up univariate ANCOVAs showed that none of the dependent variables differed significantly between the experimental and control groups (see Table 7). Estimated marginal means with 95% confidence intervals further showed that although students in the experimental group reported higher adjusted means for autonomy, relatedness, and competence than those in the control group, the confidence intervals largely overlapped, indicating that the differences were not statistically significant (see Table 8).

**Table 6:** Multivariate test for the first and the second motivation domain score as covariance.

Multivariate tests^a^								
Covariance	Effect		Value	*F*	Hypothesis df	Error df	Sig.	Partial eta squared
1st motivation domain	Group	Pillai’s Trace	0.075	2.209^b^	3.000	82.000	0.093	0.075
2nd motivation domain	Group	Pillai’s Trace	0.313	12.457^b^	3.000	82.000	0.000	0.313

**Table 7:** The dependent variables differed significantly between EG and CG.

Tests of between-subjects effects							
Source	Dependent variable	Type III sum of squares	df	Mean square	*F*	Sig.	Partial eta squared
Group	Autonomy 2	52.958	1	52.958	1.101	0.297	0.013
	Relatedness 2	143.718	1	143.718	2.888	0.093	0.033
	Competence 2	134.807	1	134.807	3.322	0.072	0.038
Group	Autonomy 3	67.640	1	67.640	1.864	0.176	0.022
	Relatedness 3	1,030.154	1	1,030.154	35.654	0.000	0.298
	Competence 3	54.732	1	54.732	1.172	0.282	0.014

**Table 8:** Group means and confidence intervals for motivation variables at Time 2 and Time 3.

Group					
Dependent variable	Group	Mean	Std. Error	95% Confidence Interval	
				Lower bound	Upper bound
Autonomy 2	CG	25.492^a^	1.067	23.370	27.614
	EG	27.133^a^	1.080	24.986	29.280
Relatedness 2	CG	25.484^a^	1.085	23.326	27.642
	EG	28.187^a^	1.098	26.003	30.371
Competence 2	CG	23.054^a^	0.980	21.106	25.003
	EG	25.672^a^	0.992	23.700	27.644
Autonomy 3	CG	29.081^a^	0.916	27.259	30.902
	EG	30.895^a^	0.927	29.052	32.738
Relatedness 3	CG	24.669^a^	0.817	23.043	26.294
	EG	31.748^a^	0.827	30.103	33.393
Competence 3	CG	27.148^a^	1.039	25.082	29.215
	EG	28.780^a^	1.052	26.689	30.871

By contrast, when the second motivation domain (Autonomy 2, Relatedness 2, and Competence 2) was entered as covariates, the multivariate effect of group was statistically significant, Pillai’s Trace = 0.313, *F*(3, 82) = 12.46, *p* < 0.001, partial *η*^2^ = 0.313 (see Table 6). This suggests that FA produced a significant overall difference in the combined dependent variables (Autonomy 3, Relatedness 3, Competence 3) after controlling for the second motivation domain. Follow-up univariate ANCOVAs revealed that only Relatedness 3 differed significantly between groups, *F*(1, 85) = 35.65, *p* < 0.001, partial *η*^2^ = 0.298, whereas Autonomy 3, *F*(1, 85) = 1.86, *p* = 0.176, partial *η*^2^ = 0.022, and Competence 3, *F*(1, 85) = 1.17, *p* = 0.282, partial *η*^2^ = 0.014, did not (see Table 7). Estimated marginal means with 95% confidence intervals confirmed EG scored substantially higher than CG on Relatedness 3, with non-overlapping confidence intervals, supporting the strong statistical effect. For Autonomy 3 and Competence 3, the overlapping intervals indicated that the observed group differences were not statistically significant (see Table 8).

Taken together, these analyses show that FA did not yield significant improvements in the second measurement point after controlling for the first motivation domain, but it did produce a robust positive effect on relatedness at the third measurement point after controlling for the second domain, while autonomy and competence showed only modest, non-significant gains.

### 4.2 Result of RQ2

Table 9 presents the descriptive statistics for the main study variables. Mean scores for achievement, autonomy, competence, and relatedness were higher in the experimental group than in the control group, suggesting a general positive association with FA. Standard deviations were similar across groups, indicating comparable variability. Additionally, A post-hoc power analysis was conducted based on the observed means for EG and CG, with sample sizes of 45 and 44, respectively. The analysis yielded a statistical power of 100%, indicating that the current sample size was sufficient to detect a moderate effect size (*α* = 0.05).

**Table 9:** Descriptive statistics of achievement, autonomy, relatedness, competence in CG and EG.

Group		Mean	Std. Deviation	Skewness statistic	Kurtosis statistic
CG	Achievement	79.77	9.815	−0.460	−0.292
	Autonomy	26.41	7.799	0.171	−0.818
	Relatedness	28.33	7.825	0.338	−0.800
	Competence	27.76	8.122	0.117	−0.501
EG	Achievement	83.02	9.915	−0.241	−0.306
	Autonomy	27.17	6.597	−0.158	−0.159
	Relatedness	29.01	6.667	0.081	−0.671
	Competence	28.70	6.785	−0.061	−1.05

**Figure 3:**
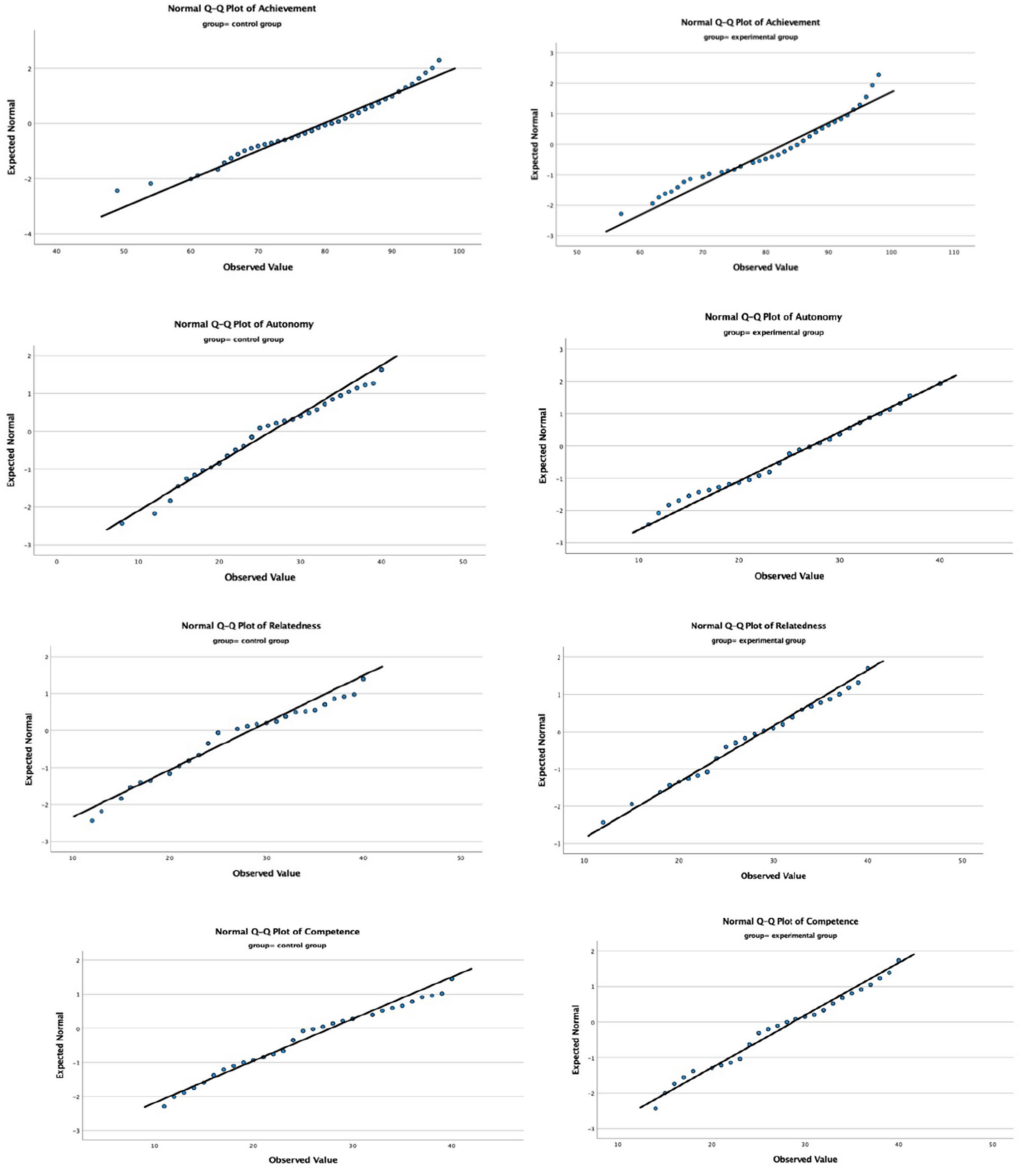
Q-Q plots of variables.

Prior to conducting multiple regression analyses, the statistical assumptions were examined. The normality of residuals was evaluated using normal Q–Q plots (see Figure 3). The points in each plot closely followed the diagonal line, indicating that the assumption of normality was reasonably met for both EG and CG. In addition, tests of multicollinearity were also conducted, with tolerance values above 0.10 and variance inflation factor (VIF) values well below the critical threshold of 10, confirming that multicollinearity was not a concern (see Table 10). Taken together, these results indicate that the assumptions for multiple regression analysis were sufficiently satisfied, allowing for subsequent analyses to be conducted with confidence.

**Table 10:** Multicollineariy of achievement, autonomy, relatedness, and competence.

Correlations						
Group			Achievement	Autonomy	Relatedness	Competence
CG	Pearson correlation	Achievement	1.000	0.033	0.043	0.088
		Autonomy	0.033	1.000	0.191	0.032
		Relatedness	0.043	0.191	1.000	0.230
		Competence	0.088	0.032	0.230	1.000
EG	Pearson correlation	Achievement	1.000	0.176	0.194	0.168
		Autonomy	0.176	1.000	0.026	−0.114
		Relatedness	0.194	0.026	1.000	0.118
		Competence	0.168	−0.114	0.118	1.000

To examine whether motivation mediated the relationship between formative assessment (FA) and achievement, multiple regression analyses were conducted separately for the EG and CG. As shown in Tables 11, 12, the regression model was not significant in the control group, *F*(3, 131) = 0.40, *p* = 0.756, with *R*^2^ = 0.009 and adjusted *R*^2^ = −0.014. This indicates that autonomy, competence, and relatedness explained virtually none of the variance in achievement in the absence of FA. By contrast, the model was significant in the experimental group, *F*(3, 128) = 4.48, *p* = 0.005, with *R*^2^ = 0.095 and adjusted *R*^2^ = 0.074, suggesting that motivation variables explained approximately 10% of the variance in achievement under FA. Effect sizes calculated using Cohen’s *f*^2^ showed a negligible effect in the control group (*f*^2^ ≈ 0.009) and a small-to-moderate effect in the experimental group (*f*^2^ ≈ 0.105).

**Table 11:** The result of multiple regression in EG and CG.

Model summary^b^					
Group	Model	*R*	*R* square	Adjusted *R* square	Std. error of the estimate
Control group	1	0.095^a^	0.009	−0.014	9.882
Experimental group	1	0.308^a^	0.095	0.074	9.543

a Predictors: (Constant), Competence, Autonomy, Relatedness.

b Dependent Variable: Achievement.

**Table 12:** The statistical significance of the result through ANOVA^a^.

Group	Model		Sum of squares	df	Mean square	*F*	Sig.
CG	1	Regression	116.091	3	38.697	0.396	.756^b^
		Residual	12,793.790	131	97.663		
		Total	12,909.881	134			
EG	1	Regression	1,222.794	3	407.598	4.476	.005^b^
		Residual	11,656.137	128	91.064		
		Total	12,878.932	131			

^a^Dependent Variable: Achievement.

b Predictors: (Constant), Competence, Autonomy, Relatedness.

Table 13 shows the contribution of each motivational variable. In the control group, none of the predictors were significant: autonomy (*β* = 0.03, *p* = 0.761, 95% CI [−0.19, 0.26]), relatedness (*β* = 0.02, *p* = 0.836, 95% CI [−0.23, 0.28]), and competence (*β* = 0.08, *p* = 0.356, 95% CI [−0.14, 0.39]). In the experimental group, however, all three predictors significantly contributed to achievement: autonomy (*β* = 0.19, *p* = 0.026, 95% CI [0.04, 0.54]), relatedness (*β* = 0.17, *p* = 0.048, 95% CI [0.00, 0.51]), and competence (*β* = 0.17, *p* = 0.049, 95% CI [0.00, 0.48]). Collinearity statistics (Tolerance > 0.90, VIF ≈ 1.0) confirmed no multicollinearity issues.

**Table 13:** Evaluating contribution of autonomy, relatedness, and competence.

Coefficients^a^											
Group	Model		Unstandardized coefficients		Standardized coefficients	Sig. (*p*)	Correlations part	95.0% Confidence Interval for B		Collinearity statistics	
			B	Std. Error	Beta (*β*)			Lower bound	Upper bound	Tolerance	VIF
CG	1	(Constant)	75.204	4.697		0.000		65.913	84.496	0.963	1.038
		Autonomy	0.034	0.112	0.03	0.761	0.026	−0.187	0.255	0.913	1.095
		Relatedness	0.027	0.130	0.02	0.836	0.018	−0.230	0.283	0.947	1.056
		Competence	0.123	0.133	0.08	0.356	0.081	−0.140	0.386		
EG	1	(Constant)	61.623	5.901		0.000		49.947	73.300	0.985	1.015
		Autonomy	0.287	0.127	0.19	0.026	0.190	0.035	0.539	0.984	1.016
		Relatedness	0.254	0.127	0.17	0.048	0.168	0.002	0.506	0.972	1.029
		Competence	0.240	0.121	0.17	0.049	0.167	0.001	0.479	0.963	1.038

a Dependent Variable: Achievement.

These findings indicate that motivational resources had little explanatory value in the absence of FA but became significant positive predictors of achievement under FA. This pattern supports a mediation model in which FA enhances students’ autonomy, relatedness, and competence, which in turn facilitates higher achievement.

## 5 Discussion

### 5.1 FA effect on EFL students’ autonomy, relatedness and competence

The findings reveal that FA had its most pronounced impact on relatedness, while its effects on autonomy and competence, though positive in direction, did not reach statistical significance. This suggests that in Chinese EFL classrooms, FA practices such as peer assessment, self-assessment, and teacher feedback are particularly effective in fostering students’ sense of connectedness and belonging. These outcomes align with Furrer and Skinner’s (2003) conceptualization of relatedness as the emotional bonds that strengthen students’ motivation to engage with learning. Students in EG may have felt closer to peers and more supported by teachers through ongoing feedback cycles, which in turn increased their willingness to persist in English learning.

This relational effect is consistent with a growing body of research emphasizing that FA enhances learning by satisfying students’ psychological need for relatedness. For instance, Lin (2025) found that self-assessment and reflection practices improved academic wellbeing by strengthening motivational resources. Similarly, Lin et al. (2025) demonstrated that online game-based FA improved learning performance through motivational incentives, particularly via social interaction. Pat-El et al. (2024) further noted that students’ perceptions of assessment for learning boosted intrinsic motivation when assessment was experienced as supportive rather than evaluative. Baines et al. (2025) echoed this in higher education, showing that authentic assessment practices enhanced motivation when aligned with students’ preference for socially meaningful learning. Collectively, these findings suggest that FA may be especially effective in collectivist contexts such as China, where peer and teacher relationships play a critical role in shaping motivation.

By contrast, autonomy and competence were not significantly affected. According to Ryan and Deci (2020), autonomy involves perceptions of volition and choice, while competence refers to feeling effective in learning tasks. The current finding suggests that FA activities did not adequately support these conditions. Students may have viewed assessment tasks as obligatory rather than self-directed, thereby limiting autonomy. Likewise, limited opportunities to monitor tangible progress may have hindered the development of competence. This interpretation aligns with Leenknecht et al. (2021), who found that FA alone may not strengthen competence unless paired with explicit goal clarification and feedback. De Jonge et al. (2025) also emphasized that process-oriented feedback, rather than frequent assessment, is crucial for reducing anxiety and strengthening competence beliefs. Similarly, Dumitru and Dragomir (2025) argue that assessment-focused pedagogy improves competence only when it provides actionable strategies for improvement.

In the present study, the lack of significant effects on autonomy and competence may reflect specific classroom dynamics. Chinese middle school students are typically accustomed to structured, teacher-centered learning in which assessments are perceived as mandatory rather than self-directed (Chen et al., 2021). As a result, formative tasks may not have been experienced as opportunities for genuine choice, limiting their capacity to foster autonomy. Additionally, although students received regular feedback, much of it may have remained evaluative rather than developmental, offering limited guidance for improvement. Without explicit benchmarks or actionable strategies, students may have struggled to track their own progress, thus constraining competence satisfaction.

Wang et al. (2024) found that FA influenced autonomy only when mediated by psychological empowerment and positive academic emotions. This aligns with Dumitru and Dragomir (2025), who highlighted the role of structured tools such as the Autonomous Learning Skills Assessment Scale in nurturing autonomy. Similarly, Jaison et al. (2025) and Yilmaz Soylu et al. (2025) showed that while digital FA can support motivation, they required sustained scaffolding, particularly through personalized feedback to effectively promote autonomy and competence. In the context of EFL learning, these findings suggest that FA should not be used merely as an evaluative tool but as a pedagogical strategy that affirms students’ efforts, encourages genuine choice, and scaffolds competence and strengthening relational bonds.

### 5.2 Autonomy as the strongest predictor of achievement

Within the FA group, autonomy emerged as the strongest predictor of EFL achievement, outperforming both relatedness and competence. This finding underscores the central role of autonomy within the SDT framework, where serves as the organizing principle for self-regulated learning, persistence and deep engagement (Ryan and Deci, 2020). Autonomy enables learners to internalize learning goals, evaluate their own progress, and sustain motivation in the absence of external control, skills essential for long-term academic success. This outcome corroborates findings in other EFL contexts. For example, Belinda (2020), Alamer and Al Khateeb (2023) and Yan and Chiu (2023) all reported that autonomy-supportive environments, those offering meaningful choice, opportunities for goal setting, and scaffolding for self-regulation, were the most consistent drivers of language achievement. In our study, regression analysis similarly revealed that FA enhanced academic performance primarily by supporting students’ perceived agency in learning.

FA practices such as peer and self-assessment, reflective feedback, and opportunities for learner voice directly contribute to autonomy by transferring partial control of learning to the students. These practices reduce the traditional teacher-dominant structure and instead foster conditions in which students actively monitor, evaluate, and adjust their performance. As De Neve et al. (2022) emphasize, the diversifying feedback sources (self, teacher, peer) enables learners to internalize standards and assume greater responsibility for improvement. This autonomy-driven ownership over learning processes is likely to yield stronger, more sustained achievement outcomes than external control or passive instruction. Moreover, recent innovations in digital and gamified FA offer personalized feedback loops and adaptive task selection, further reinforcing learners’ sense of autonomy (Yilmaz Soylu et al., 2025). These tools support choice, self-pacing, and real-time monitoring, all of which enhance autonomy. Alabidi et al. (2022) likewise showed that non-traditional digital platforms, although modest in their effects, could shift learners’ motivational orientation by providing meaningful, self-directed engagement opportunities.

While autonomy stood out as the most influential factor, this does not negate the motivational value of relatedness and competence. Indeed, FA strategies can also address these needs, as evidenced by mobile-assisted feedback systems that simultaneously enhance all three SDT components (Nikou and Economides, 2021). However, in our context, the effects of competence were limited. This may be attributed to the relatively short duration of the intervention and the predominantly evaluative nature of the feedback students received. As suggested by De Jonge et al. (2025) and Dumitru and Dragomir (2025), competence development depends on feedback that is process-oriented, actionable, and clearly aligned with learning goals. In the absence of such detailed scaffolding, students may have struggled to perceive real progress, even when cognitively engaged. Similarly, the influence of relatedness, although evident in earlier analyses, may not translate as directly to achievement outcomes as autonomy does. Relatedness fosters a supportive emotional climate, but autonomy enables learners to initiate, sustain, and direct their own efforts, an especially vital trait in test-driven environments like Chinese EFL classrooms, where individual responsibility and academic outcomes are tightly linked.

## 6 Conclusion

This study demonstrates that FA is positively associated with EFL learners’ motivation and achievement, especially through supporting relatedness. The findings suggest that FA does not directly drive achievement performance but operates by satisfying basic psychological needs, thereby refining the explanatory power of SDT in language education. Building on this, the current model extends SDT to the domain of classroom assessment by conceptualizing FA not merely as an instructional technique but as a motivational process that interacts with learners’ psychological needs to influence achievement. From a pedagogical perspective, these results highlight the importance of embedding timely feedback, peer or self-assessment, and reflective opportunities into instruction to cultivate student-centered classrooms. However, several limitations should be acknowledged. The study was conducted in a single middle school with a relatively small sample over an eight-week period, which restricts generalizability and limits the ability to capture long-term effects. Contextual and cultural factors, such as the exam-driven and teacher-centered nature of Chinese classrooms, may also have constrained the autonomy and competence, related to the impact of FA. Moreover, teacher bias cannot be ruled out, as the quality of feedback and peer assessment may have varied across lessons. Future research should employ larger, multi-site, and longitudinal designs to determine whether these findings hold across diverse age groups, disciplines, and cultural contexts. Further investigation can also explore how FA affects affective variables such as anxiety, confidence, and learning satisfaction. Such studies will not only deepen the practical implications for EFL pedagogy but also extend the theoretical application of SDT to classroom assessment contexts.

## Data Availability

The data that support the findings of this study are available from the corresponding author upon reasonable request.
