# Supplementary material for: Investigating the effects of formative assessment on EFL students’ achievement and motivation: a Self-Determination Theory perspective
Source: Front Psychol. 2025 Dec 12;16:1664871. doi: 10.3389/fpsyg.2025.1664871 (PMC12741153; doi:10.3389/fpsyg.2025.1664871)
Supplement: Supplementary file 1 [file Table_1.docx]

# Appendix A Overview of Formative Assessment Practices Integrated into the Intervention Lessons

| Lesson | Unit Topic | Skill Focus | Formative Assessment Practice | Tool Used | Frequency | Student Output |
| --- | --- | --- | --- | --- | --- | --- |
| 1 | It’s raining! | Reading | Peer assessment with rubric on reading comprehension | Peer rubric | Once | Oral + rubric |
| 2 |  | Writing | Self-assessment checklist for paragraph writing | Self-checklist | Twice | Paragraph draft |
| 3 |  | Summary | Reflective summary and group sharing | Reflection worksheet | Once | Summary reflection |
| 4 | Is there a post office near here? | Reading | Teacher feedback on comprehension questions | Feedback sheet | Once | Answer sheet |
| 5 |  | Writing | Student-generated questions and peer scoring | Peer scoring form | Once | Peer-marked Qs |
| 6 |  | Summary | Summary writing with self-reflection journal | Reflection journal | Once | Written summary |
| 7 | What does he look like? | Reading | Group discussion and oral peer feedback | Discussion log | Once | Group talk |
| 8 |  | Writing | Checklist-based self-evaluation of writing | Writing checklist | Twice | Improved draft |
| 9 |  | Summary | Post-task reflection on character description | Reflection prompt | Once | Reflection notes |
| 10 | I’d like some noodles. | Reading | Interactive quiz with immediate feedback | Quiz platform | Once | Quiz score |
| 11 |  | Writing | Self-assessment on recipe writing using rubric | Writing rubric | Once | Recipe writing |
| 12 |  | Summary | Peer review of menu design presentation | Peer feedback form | Once | Peer comments |

# Appendix B Lesson Plan (example)

Formative assessment strategy integration lesson plan (Sample)

Unit 7: It’s raining! (lesson 1)

Main focus: Reading

| Stages | Objectives | Activity & FA practice | FA tool |
| --- | --- | --- | --- |
| Pre-reading | Activate schema and vocabulary | Students complete picture-description and sentence-building task based on weather scenes. Peers assess sentence accuracy and clarity. | Peer rubric checklist |
| While-reading | Identify structure and style of informal writing | Students analyse postcards (Textbook 2b). Use questioning strategy (e.g., “What would you write in a postcard?”). Peers share and give feedback on ideas. | Guided questions + peer discussion |
| Post-reading | Scanning and memory recall for gist and detail | “No looking!” challenge to reconstruct key facts from memory (Textbook 2c). Students complete chart individually, then exchange and **peer-check.** | Chart template + peer feedback |
| Reflection | Monitor learning progress | Students complete brief self-assessment reflection: “What did I learn about describing weather & activities?” | Reflection form |

Unit 7: It’s raining! (lesson 2)

Main focus: writing

| Stages | Objectives | Activity & FA practice | FA tool |
| --- | --- | --- | --- |
| Pre-writing | Build prediction and vocabulary awareness | “What do I know?” vocabulary activity. Students predict missing words, then **compare responses in pairs and give corrections**. Teacher gives immediate feedback. | Peer correction, teacher oral feedback |
| Planning | Imagine and personalise writing content | Guided visualisation + paired sharing on imagined vacation (3b). Peers listen and **ask clarifying questions** using a checklist. | Imagination checklist + oral feedback |
| Drafting | Produce postcard-style writing | Write postcard (3c). Students then **exchange drafts**, give peer feedback on clarity, tone and structure using a writing rubric. Teacher gives summary feedback. | Writing rubric (peer + teacher) |
| Reflection | Encourage metacognitive awareness | Students submit postcard draft and **self-evaluate using criteria**: “What did I do well? What can I improve?” Reflection added to writing portfolio. | Self-evaluation sheet + portfolio |
